# EXPLORING USE OF COMMUNITY SUPPORT SERVICES BY BLACK FAMILY DEMENTIA CAREGIVERS: FACILITATORS AND BARRIERS

**DOI:** 10.1093/geroni/igae098.3389

**Published:** 2024-12-31

**Authors:** Florence Johnson, Sheria Robinson-Lane

**Affiliations:** University of Michigan, Ann Arbor, Michigan, United States; University of Michigan, Ann Arbor, Michigan, United States

## Abstract

In this qualitative study, we delve into the factors influencing community support services (CSS) utilization, including respite care and support groups. We conducted focus group discussions to uncover the barriers and facilitators that lead individuals to engage with or abstain from these services. Analyzing these factors will provide insights to enhance the effectiveness of CSS and its impact on the community. Our focus group involved six current or former Black family caregivers of persons with dementia (PWD). The virtual meeting, lasting approximately 1.5 hours, collected information on community support use, dementia care knowledge, and self-care practices. Transcriptions were thematically analyzed using the Rigorous and Accelerated Data Reduction (RaDAR) method—a team-based strategy to extract relevant data for specific project deliverables. Four key themes emerged to aid caregivers in managing their responsibilities: Education on Dementia Stages: Understanding different stages of dementia helps caregivers effectively manage challenging behaviors. Empowerment through Dementia Subtypes: Knowledge of various dementia subtypes enables caregivers to seek support groups for guidance. Respite Care Access: Learning about and accessing respite care prevents burnout and promotes well-being. Emotional Support: Addressing guilt provides emotional well-being for caregivers. As the older adult population grows, dementia caregiving becomes a critical public health issue. Family members caring for PWD in the community face significant challenges, emphasizing the importance of effective support services.

